# Meta-analysis data of 104 renewable mini-grid projects for rural electrification

**DOI:** 10.1016/j.dib.2021.106739

**Published:** 2021-01-09

**Authors:** A. Serasu Duran, Feyza G. Sahinyazan

**Affiliations:** a Haskayne School of Business, University of Calgary, AB, Canada; b Beedie School of Business, Simon Fraser University, Vancouver, BC, Canada

**Keywords:** Rural electrification, Renewable energy, Mini-grid, Meta-analysis

## Abstract

The data presented here contains project-level details on 104 renewable energy mini-grid projects installed for rural electrification across the globe; a subset of which is used to derive evidence-based empirical insights on the drivers of project success and cost in the article “An Analysis of Renewable Mini-Grid Projects for Rural Electrification” [1]. To the best of our knowledge, this is the first meta-collection of micro-level data on rural mini-grid installations. In addition, the literature search and the inclusion criteria of the studies used in the meta-analysis is reported, along with a complete list of sources, which can be utilized directly by other researchers and practitioners to reproduce or expand the database according to their own criteria and use it in further studies. Finally, the supplemental material in [2] includes the Stata code and output that can be used to reproduce the meta-analysis results in [1].

## Specifications Table

**Table:** 

Subject	Renewable Energy, Sustainability and the Environment
Specific subject area	Renewable energy mini-grid projects used in rural electrification
Type of data	Text data Excel spreadsheet Supplemental material
How data were acquired	Data is collected from the primary project documentation included in the meta-analysis by manual encoding. The primary documentation includes multiple types of publications and media, and were identified in guidance of the PRISMA protocol [3]. Search terms are the combinations: renewable, rural, mini-grid, off-grid, electrification, project, pilot.
Data format	Raw Analyzed
Parameters for data collection	The criteria used for inclusion of projects into the data set are: The projects within the dataset must be rural, and renewable, i.e., they must include at least a single renewable generation source and not within the reach of existing national grids. The installation should have commenced no later than 2018 in order to have at least a year's worth of operation. The installation should have commenced no earlier than 1995 due to the rapidly changing costs and characteristics of renewable technologies. The capacity and the ownership details must be available. The project must support at least a population of 5, but not exceed 25 MW of total capacity.
Description of data collection	The primary data sources were identified as a result of an extensive and systematic search in guidance of the PRISMA protocol [3]. Project documentations were screened for eligibility by two independent authors. Data regarding each project was triangulated and cross-verified from at least two sources, and extracted according to the inclusion criteria (see ‘Experimental designs, Materials, and Methods’ for further details). A systematic coding form was used to record relevant information from each project.
Data source location	A complete list of the primary sources can be found in the repository, in the file named ‘Project References.pdf’.
Data accessibility	Public repository [2] Repository name: Mendeley Data Data identification number: doi:10.17632/s3wm2sgjjb.2 Direct URL to data: http://dx.doi.org/10.17632/s3wm2sgjjb.2
Related research article	A. Serasu Duran, Feyza G. Sahinyazan, An Analysis of Renewable Mini-Grid Projects for Rural Electrification, Socio-Economic Planning Sciences (SEPS), Forthcoming. DOI: 10.1016/j.seps.2020.100999

## Value of the Data

• The outcomes and the lessons learned from renewable mini-grid projects remain highly compartmentalized, making it significantly challenging to derive evidence-based insights on clean rural electrification for investors and practitioners. This dataset clearly reports and presents micro-level data for 104 such projects across the globe, along with the information necessary to replicate and expand both the data collection and a related meta-analysis.
• Researchers, practitioners, investors and policymakers looking to gain evidence-based understanding on renewable rural electrification can directly benefit from this data.
• The mini-grid project database can be used to duplicate or expand the meta-analysis to derive additional insights. Furthermore, the data collection methodology can be replicated and the project documentation references can be utilized to create new or modified databases according to the needs of other researchers.
• The data can be used to test hypotheses associated with mini-grid projects in remote zones, to derive quantitative evidence on the best success-oriented measures that can be implemented at the design stage of mini-grid projects, or be fed into decision-making or optimization models to calibrate them and to inform the feasibility of the output generated by such models.

## 1 Data Description

Four files are available in the online repository [2]: *projectdata.xlsx, datadescription.pdf, Project References.pdf, Stata Output.txt*. In *projectdata.xlsx* the detailed micro-level project data on 104 rural renewable electrification projects is presented. Each row corresponds to an individual mini-grid project, with project-related variables presented in the columns. The descriptions of the variables are provided in *datadescription.pdf.*

Table 1 provides the encoding values for the variables.

**Table 1:** Variable encoding values.

*Variable*	Encoding	*Variable*	Encoding
*Pid*	Integer	*Hydro*	Binary; 0=None, 1=Included
*Country*	Text	*Biomass*	Binary; 0=None, 1=Included
*Class*	Text; categorical	*Hybrid*	Binary; 0=No, 1=Yes
*Ownership*	Text; categorical	*DieselSizeKW*	Decimal; “n/a” if no Diesel installed
*YearBuilt*	Integer	*SolarSizeKW*	Decimal; “n/a” if no Solar installed
*YearLast*	Integer	*WindSizeKW*	Decimal; “n/a” if no Wind installed
*StorageTech*	Text; categorical	*HydroSizeKW*	Decimal; “n/a” if no Hydro installed
*Storage*	Binary; 0=None, 1=Included	*BiomassSizeKW*	Decimal; “n/a” if no Biomass installed
*StorageSizeKWh*	Decimal	*Project Cost per Capita (PCCC)*	Decimal
*Demand Management (DM)*	Binary; 0=None, 1=Included	*Total system capacity per capita (SCPC) (kW)*	Decimal
*Renew*	Decimal	*Success*	Binary; 0=Failed, 1=Successful
*Diesel*	Binary; 0=None, 1=Included	*Cost (2020USD)*	Integer
*Solar*	Binary; 0=None, 1=Included	*CostEstimated*	Binary; 0=Actual, 1=Estimated
*Wind*	Binary; 0=None, 1=Included	*Population*	Integer

**Fig. 1:**
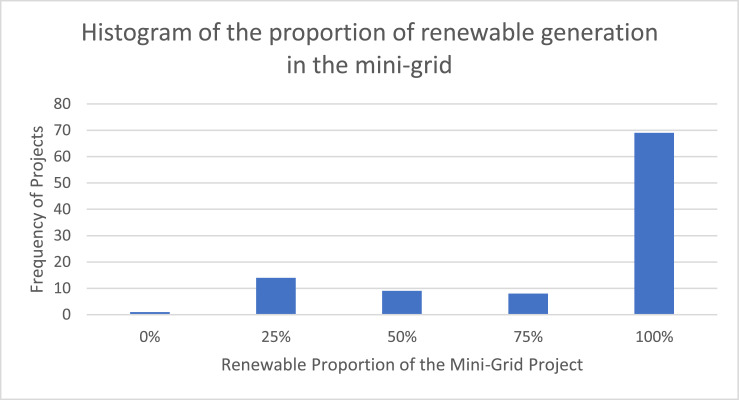
Histogram of the proportion of renewable generation in the mini-grid.

Fig. 1 visualizes the proportion of renewable generation that is included in the capacity mix of each mini-grid project. More than two thirds of the projects are entirely based on renewable generation.

**Fig. 2:**
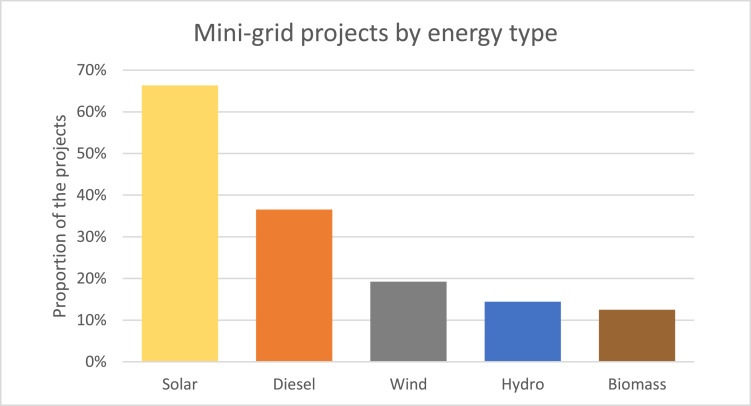
Distribution of mini-grid projects by their energy source type.

Fig. 2 depicts the representation of the various types of renewable energy sources across the projects in the dataset. The most popular renewable energy source is Solar, which is included in the generation mix of about two thirds of the projects. Wind follows solar as the second most popular renewable source type with a huge gap, as only one fifth of the projects have a wind generation component. In contrast, Diesel as a back-up generation source is included in about one third of the projects. Overall, In 39.4% of the mini-grid projects more than one energy type is used in tandem. 90.2% of the hybrid projects have a diesel component as a back-up energy source.

**Fig. 3:**
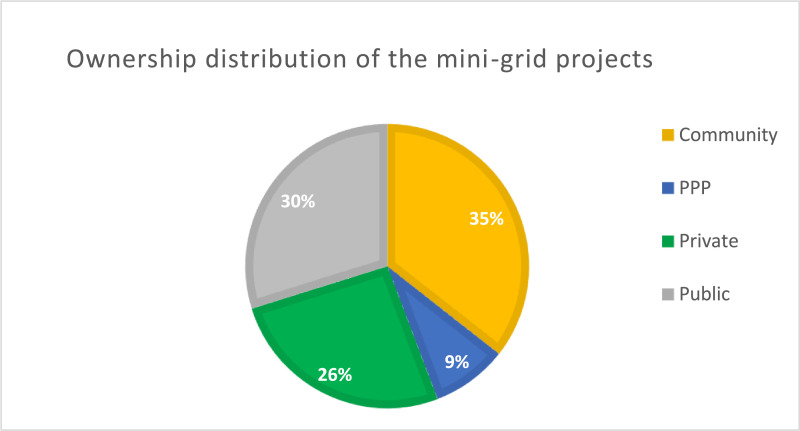
Distribution of mini-grid projects by ownership structure.

Fig. 3 compares the various types of ownership for the mini-grids among the four different types (Community ownership, Private ownership, Public ownership, Public-Private Partnership).

In Table 2, the installation years of the projects are summarized. The dataset represents more projects installed in the last decade, with the majority of projects installed between 2011 and 2015.

**Table 2:** Distribution of mini-grid projects by years.

Years	Number of mini-grid projects installed
1995–2000	3
2001–2005	8
2006–2010	20
2011–2015	48
2016–2018	25

**Fig. 4:**
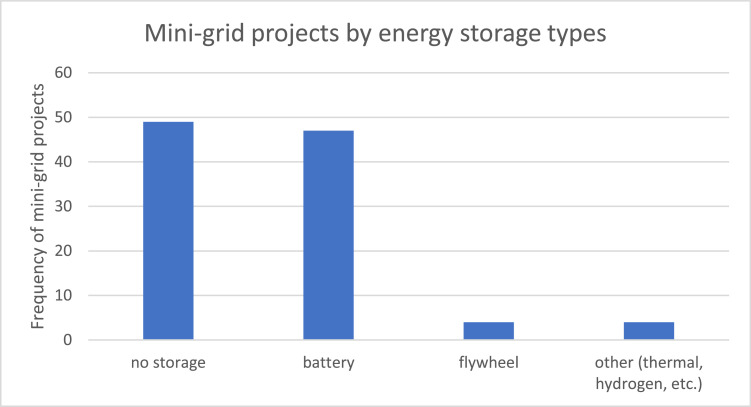
Distribution of mini-grid projects by the energy storage types.

Fig. 4 compares the type of energy storage present in the mini-grid installations. Close to half of the projects have no storage, and the other half predominantly uses batteries for conserving energy.

In terms of the summary of the categorical variables, we find that 52.9% of the projects have a storage component and 15.4% of the projects have a demand management component. Furthermore, 86.5% of the projects were found to be successful as of 2018. The summary statistics for non-categorical variables are provided in Table 3.

**Table 3:** Summary statistics of the numerical variables in the dataset.

*Variable*	Min	Max	Mean	std. dev
*StorageSize* (KWh)	0	8064	487.39	1345.39
*DieselSize* (KW)	5	18,340	2227.39	4137.14
*SolarSize* (KW)	1	1400	192.16	339.75
*WindSize* (KW)	0.35	11,000	1481.38	2668.71
*HydroSize* (KW)	5	1350	284.67	430.16
*BiomassSize* (KW)	6	250	69.58	71.13
*PCCC* (2020USD)	3.06	10.97	6.19	1.97
*SCPC* (KW)	0.0006	6.176471	0.52	1.12
*Cost (2020USD)*	6906	71,000,000	4069,257	9337,421
*Population*	8	84,229	4828.30	13,092.30

In *Project References.pdf,* the corresponding primary references for each project are listed, to aid in verification of the collected data and provide transparency in data collection. In *Stata Output.txt,* the Stata code syntax and the original regression output for replicating the meta-analysis is provided.

## 2 Experimental Design, Materials and Methods

An extensive meta-analysis of existing mini-grid installations and pilot projects in rural areas across the world was conducted, and a cross-sectional dataset is collected from the 104 projects included in the meta-analysis.

i. **Data Collection Strategy**

The data related to each project is manually encoded from a vast collection of primary documentation which includes multiple types of publications and media. These primary data sources include scientific publications, reports and databases from governments, public utilities, regulators and rural electrification agencies, grey literature such as NGO, donor and development agency reports, case studies, portfolios of private developers, official company press releases and news articles. The primary sources were identified as a result of an extensive and systematic search in guidance of the PRISMA protocol [3]. Project documentations were screened for eligibility by two independent authors. Publications that only specify macro-level statistics were excluded in favor of data sources that specify project details in the community and at the micro-level. Data regarding each project was triangulated and cross-verified from at least two sources. The project information relevant to the hypotheses of the meta-analysis was manually coded from these primary sources using a standardized spreadsheet form.

The target search terms used to identify relevant projects are the combinations of terms: renewable, rural, mini-grid, off-grid, electrification, project, pilot. The 104 eligible projects in the database were selected according to the following inclusion criteria:

A. The focus of the meta-analysis is rural and renewable mini-grid projects. Therefore, installations that have no renewable generation source and that are located in urban areas reached by existing national grids are excluded.
B. Since measuring success factors is one of the major goals of the meta-analysis, only the projects that have been commissioned at and before the year 2018 are included in order to have at least a year's worth of operation. Due to the rapidly changing costs and characteristics of renewable technologies, the projects that were commissioned before the year 1995 were excluded.
C. Projects that were missing capacity and ownership details were excluded, as these two variables are critical to the meta-analysis objectives.
D. To limit the attention to ‘mini-grids’, projects that are included support at least 5 households and are smaller than 25 MW of total capacity.

Researchers that wish to employ this dataset are encouraged to screen the list of primary references to include or exclude further projects according to their own criteria.

ii. **Meta-analysis**

The empirical analysis was conducted using the statistical software Stata 16.1. Descriptive methodologies are used alongside regression analysis, for which the code and the output is provided in the repository along with the dataset.

**Fig. 5:**
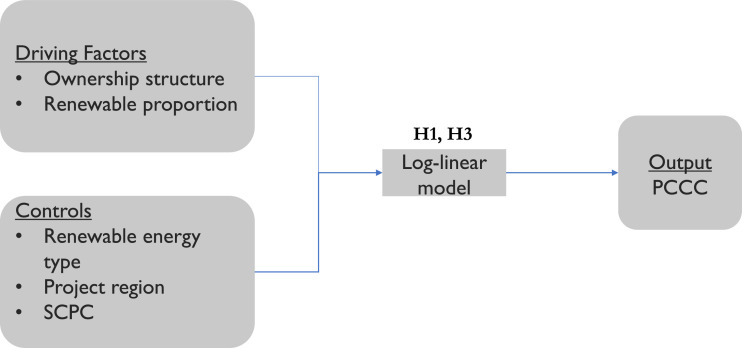
Project cost estimation model.

**Fig. 6:**
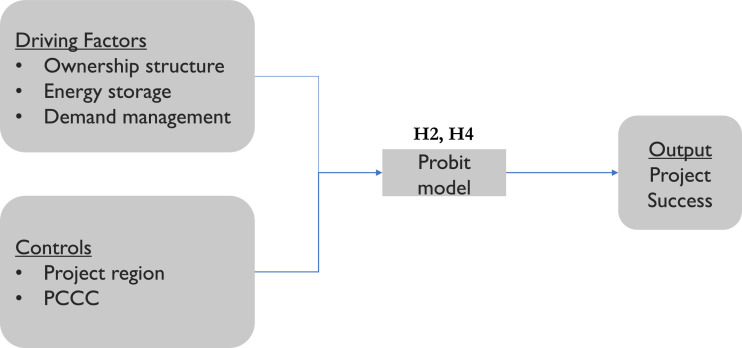
Project success estimation model.

The factors affecting the cost of a mini-grid project were determined by an ordinary least-squares regression model, and the success factors were investigated by a probit model. Figs. 5 and 6 show the driving factors and the output for both of these models along with the controls used and the hypotheses tested.

Hypothesis 1 (H1) checks the effect of renewable proportion in the mini-grid on project costs per capita, whereas Hypothesis 3 (H3) looks at the effect of the ownership structure. We find support for both of our hypotheses related to project costs. The coefficient estimates show significant negative coefficients for private-based (*p*<0.05) and community-based (*p*<0.1) projects, meaning that these ownership models will result in lower total project costs per beneficiary compared to publicly owned mini-grids. On the other hand, the coefficient estimate for the renewable proportion (*p*<0.05) has a significant and positive coefficient, supporting (H1), and implying that increasing the share of renewable sources in the generation mix of the mini-grids will raise the project costs per capita. Hypothesis 2 (H2) checks whether energy storage or demand management has an impact on project success, while Hypothesis 4 (H4) looks at the effect of ownership structure. We find a significant positive relationship (*p*<0.05) between the community-based ownership model and project success, validating (H4). However, (H2) is only partially supported. Energy storage is found to have a significant positive effect on project success (*p*<0.05), but there is no significant effect of demand management technologies.

## Ethics Statement

Not applicable.

## CRediT Author Statement

**A. Serasu Duran:** Conceptualization; Data curation; Writing - Original draft preparation; **Feyza G. Sahinyazan:** Visualization; Validation; Application of Statistical Techniques.

## Declaration of Competing Interest

The authors declare that they have no known competing financial interests or personal relationships which have or could be perceived to have influenced the work reported in this article.
